# Assessment of knowledge about biobanking among healthcare students and their willingness to donate biospecimens

**DOI:** 10.1186/s12910-017-0195-8

**Published:** 2017-05-02

**Authors:** Leena Merdad, Lama Aldakhil, Rawan Gadi, Mourad Assidi, Salina Y. Saddick, Adel Abuzenadah, Jim Vaught, Abdelbaset Buhmeida, Mohammed H. Al-Qahtani

**Affiliations:** 1 Department of Dental Public Health, Faculty of Dentistry, King Abdulaziz University, P.O. Box 80209, Jeddah, 21589 Saudi Arabia; 2 College of Public Health and Health Informatics, King Saud Bin Abdulaziz University for Health Sciences, P.O. Box 22490, Riyadh, 11426 Saudi Arabia; 3 Center of Excellence in Genomic Medicine Research, King Abdulaziz University, P.O. Box 80216, Jeddah, 21589 Saudi Arabia; 4 Center of Innovation in Personalized Medicine, King Abdulaziz University, P.O. Box 80216, Jeddah, 21589 Saudi Arabia; 5 Department of Biology, Faculty of Science, King Abdulaziz University, Jeddah, Saudi Arabia; 6 International Society for Biological and Environmental Repositories, Vancouver, BC V5Z 1B3 Canada; 7 Biopreservation and Biobanking, Kensington, MD 20895 USA

**Keywords:** Biobank, Survey, Willingness to donate, Knowledge, Biospecimens, Medical students, Precision medicine

## Abstract

**Background:**

Biobanks and biospecimen collections are becoming a primary means of delivering personalized diagnostics and tailoring individualized therapeutics. This shift towards precision medicine (PM) requires interactions among a variety of stakeholders, including the public, patients, healthcare providers, government, and donors. Very few studies have investigated the role of healthcare students in biobanking and biospecimen donations. The main aims of this study were (1) to evaluate the knowledge of senior healthcare students about biobanks and (2) to assess the students’ willingness to donate biospecimens and the factors influencing their attitudes.

**Methods:**

A cross-sectional study was conducted among senior healthcare students at King Abdulaziz University (KAU), Saudi Arabia. The data were obtained using a self-administered questionnaire in English. In addition to the respondents’ biographical data section, the questionnaire assessed the respondents’ general knowledge about biobanking, the factors influencing their willingness to donate biospecimens to biobanks and their general attitudes towards biomedical research.

**Results:**

A total of 597 senior healthcare students were included in the study. The general knowledge score was 3.2 (±1.6) out of 7. Only approximately 44% and 27% of students were aware of the terms “Human Genome Project” (HGP) and “biobank,” respectively. The majority of the students (89%) were willing to donate biospecimens to biobanks. Multiple factors were significantly associated with their willingness to donate, including their perceived general health (*p* < 0.001), past experience with both tissue testing (*p* < 0.04) and tissue donation (*p* < 0.001), biobanking knowledge score (*p* < 0.001) and biomedical research attitude score (*p* < 0.001). The main reasons for students’ willingness to donate were advancement of medical research and societal benefits, whereas misuse of biospecimens and confidentiality breaches were the main reasons for a reluctance to donate.

**Conclusion:**

Despite their strong willingness to donate biospecimens, students exhibited a notable lack of knowledge about biobanking and the HGP. To expedite the transition towards PM, it is highly recommended to enhance healthcare curricula by including more educational and awareness programmes to familiarize students with OMICs technologies in addition to the scope of research and clinical applications.

## Background

One of the major outcomes of the Human Genome Project (HGP) is the advancement of biobanks, which are collections of biospecimens from patients and volunteer donors combined with their personal information, clinico-pathological features and even research data [1, 2]. In the post-genomic era, biospecimen collection is becoming the main mechanism of delivering personalized diagnostics and tailored individualized therapeutics [3, 4]. Therefore, biobanks are considered the primary resource that will help shape the future of human health through precision medicine (PM). Briefly, PM can be defined as the right treatment for the right patient at the right time, every time. To be implemented efficiently, PM relies mainly on elucidating the dynamic interplay between the environment (living, non-living), lifestyle and social well-being and an individual’s genomic make up in both health and disease status. Therefore, improving our understanding of the environment and lifestyle components, their digitalization, and especially assessing their contribution either a prevention or risk factor is crucial to developing customized disease-specific and/or public health strategies. Therefore, implementing standard operating procedures (SOPs) for sampling, collecting and processing biospecimens as well as considering the related bioethics and principles of responsible data sharing are thus paramount to attaining effective prevention and PM.

The current shift towards precision medicine (PM) requires comprehensive interactions between a variety of stakeholders involved in biobanking, including the public, patients, healthcare providers, the government, and donors [5, 6]. Support, understanding and collaboration between these stakeholders are key to the survival and proper functioning of biobanks. Several studies have described the role of the public [7, 8] and patients [9–11] in biobanking and tissue donation, but very few studies have investigated the role of healthcare professionals.

Public support, understanding and active involvement are crucial to the survival of biobanks and genetic research in general. Therefore, a suitable assessment of the public’s knowledge and attitudes can provide researchers with information about how to approach potential donors and seek their approval [7]. In addition, depending on whether the samples received are obtained from living or deceased donors, the collection of biospecimens requires consent from the donor or the donor’s relatives, respectively. A study conducted in Sweden that assessed the willingness of the general public to donate tissue samples concluded that 78% agreed to donate and store biospecimens for subsequent research use [7]. Researchers from Italy indicated that 86% of individuals approved of sample donation for research purposes [8].

Despite these positive attitudes towards sample donation, there is still limited understanding of the definition, role, importance, scale, and governance of biobanks and their contributions to medical research, both globally and regionally [12–15]. Several factors have been demonstrated to affect willingness to participate in biomedical research and/or donate biospecimens, including age [14, 16–19], education [1, 7, 8, 20], and concerns about lack of confidentiality [21]. In addition, religious beliefs and cultural trends have been reported to be influential factors [22, 23].

Ensuring continuous development and innovation in PM and biomedical research requires increased biobanking knowledge, training and continuous education of healthcare professionals, including undergraduate and graduate students, who are the future leaders of the field [24, 25]. A better understanding of healthcare students’ baseline knowledge of biobanks and their willingness to donate biospecimens is therefore essential. Very few studies have assessed knowledge about biobanks and willingness to donate biospecimens among healthcare workers, both in general and in Saudi Arabia in particular. The main objective of the current study was to assess the knowledge of senior healthcare students about biobanks and their willingness to donate biospecimens, with a focus on the main factors that might influence their knowledge, attitudes and practices in the PM era. This study specifically targets senior students at King Abdulaziz University (KAU) enrolled in the Faculties of Medicine, Dentistry, Pharmacology and Medical Technology.

## Methods

### Study design & participants

This cross-sectional study was conducted among healthcare students at the largest University in Saudi Arabia, King Abdulaziz University (KAU). KAU has more than 40,000 students (2015/2016) enrolled in 18 faculties within three main areas: medicine, science, and humanities. All senior students enrolled in the medical stream including the Faculties of Medicine, Dentistry, Pharmacology and Medical Technology were targeted in this study. This study focused on senior students who were about to graduate at the end of the 2015/2016 academic year. These students were targeted due to their overall collective knowledge that has been accumulated during their medical studies and their upcoming involvement as future healthcare providers in Saudi Arabia.

### Questionnaire

The data were collected using structured, self-administered questionnaires in the English language. The design of the questionnaire was based on surveys used in previously published studies [8, 9, 26], and the questionnaire was enriched with additional questions to collect useful supplementary information. Questionnaires were randomly distributed and collected from students of each faculty. The investigator explained the purpose and significance of the study and informed students that participation in the study was voluntary and that all data will be anonymous and confidential. Ethical approval was obtained from the Research Ethics Committee at KAU Hospital (Ref. number: 106-15), and special permission to conduct the survey was obtained from each faculty. The three sections of the questionnaire were about (1) personal information and the general heath background of each participant, (2) biobanking knowledge, and (3) biomedical research attitudes.

#### Participant biodata and general health status section

This descriptive section collected data about participant socio-demographic characteristics and general health, including age, gender, faculty, school year, grade-point average (GPA; out of 5), marital status, number of children and general health status. In addition to inquiring about the health history of the students, including inherited diseases and/or hospitalization, this survey recorded their previous experience with blood or organ donation, genetic testing, and/or experience with participation in biomedical research.

#### Biobanking knowledge questionnaire section

This section consisted of two parts. The first included 7 main items that evaluated the general knowledge of the participants about biobanking and biospecimens and whether he/she knew about the following: HGP or biobanking terms, the definition of biobanking, the purpose of collecting biospecimens, the concept of consent, confidentiality, and the SOPs required for biospecimen collection.

The second part assessed the students’ willingness to donate biospecimens to biobanks to perform biomedical research. It included questions about whether the participants were willing to donate tissue to biobanks, which specific tissue they were willing to donate (i.e., saliva, urine, blood, buccal swabs, toenails, hair, their own excess surgical tissue, or deceased family members’ organs or tissues) and the reasons for their willingness or unwillingness to donate.

#### Biomedical research attitudes questionnaire section

This section focused on the Research Attitudes Questionnaire (RAQ), which is a validated questionnaire used to assess general attitudes towards biomedical research [26]. It is composed of 11 items listed on a 5-point Likert scale, with scores ranging from 1, “Strongly disagree”, to 5, “Strongly agree”. A total score was generated by summing all individual items, and higher scores indicate more positive attitudes.

### Statistical analysis

The main outcomes (dependent) variables in this study were 1) knowledge about biobanking and 2) willingness to donate tissue to biobanks for biomedical research purposes. The willingness to donate was calculated as a binary variable (Yes/No) based on whether the respondent was willing to donate at least one of the specified tissues. For each of the 7 knowledge questions, a score of 1 was assigned if the participant gave a correct answer, and a score of 0 was assigned if the participant gave an incorrect answer. The percentage of participants who gave a correct answer for each knowledge question was calculated. In addition, for each participant, a total knowledge score was calculated by summing across questions, with scores ranging from a minimum of 0 to a maximum of 7. The students’ attitude towards biomedical research was measured using the RAQ. A total attitude score was generated based on the responses to each attitude item being rated on a 5-point Likert scale as follows: 1 “Strongly disagree”, 2 “Disagree”, 3 “Neutral”, 4 “Agree”, and 5 “Strongly agree”. A total attitude score was calculated for each student by summing across items, with total scores ranging from a minimum of 5 to a maximum of 55. Seven items were positively worded, and 4 were negatively worded. All negatively worded items were reversed such that a higher numbered response on the Likert scale would represent positive attitudes.

Categorical data were described using frequencies and percentages, whereas continuous data were described using means and standard deviations. The associations between predictors and willingness to donate were tested using the chi-squared test. The associations between knowledge and attitude scores and the willingness to donate were tested using the *t*-test. The significance level was set at 0.05. All statistical analysis was performed using STATA version 13 (StataCorp, College Station, Texas, USA).

## Results

The questionnaire was completed by 597 of a cohort of 693 students, yielding a response rate of 86%. The socio-demographic, health-related and biobanking-related characteristics of the study population are summarized in Table 1. Females represented 61% of the cohort. Students attended the following faculties: Medicine (39%), Dentistry (20%), Pharmacology (13%) and Medical Technology (28%). The majority of respondents had a B grade average (53%). Regarding their health status, 38% reported excellent health status, whereas very few reported fair/poor health (4%). Nine percent (9%) of students reported being diagnosed with a chronic disease, 36% reported being hospitalized and 35% reported inherited diseases in their families. Approximately 91% have had a blood test and 28% have donated blood, whereas 16% have undergone a tissue test, and only 2% had already donated tissue. About half the students (45%) have been involved in medical research.

**Table 1:** Characteristics of the study population

Socio-demographic & health-related variables	*N* = 597	%
Gender		
Male	229	39
Female	353	61
Marital Status		
Married	70	12
Non-married	511	88
Faculty		
Medicine	228	39
Dentistry	116	20
Medical technology	164	28
Pharmacology	75	13
GPA		
A	183	33
B	290	53
C/D	78	14
General Health		
Excellent	180	31
Very good	220	38
Good	160	28
Fair/Poor	20	4
History of Chronic disease		
No	528	91
Yes	51	9
Family history of inherited disease		
No	369	65
Yes	200	35
Previous hospitalization		
No	371	64
Yes	208	36
Biobanking-related variables	*N*	%
Previous genetic testing		
No	552	95
Yes	29	5
Previous blood testing		
No	51	9
Yes	531	91
Previous tissue testing		
No	486	84
Yes	92	16
Previous blood donation		
No	415	72
Yes	163	28
Previous tissue donation		
No	565	98
Yes	13	2
Involvement in medical research		
No	319	55
Yes	257	45

Table 2 shows the students’ responses to the biobanking knowledge questions. The mean knowledge score was 3.6 ± 1.8 out of 7. Only 40% of students have heard of the HGP, whereas only 27% have heard of the term “biobanks”. When asked about the purpose of biobanks, 59% correctly responded that it was to collect and store biospecimens for diagnosis, treatment and research purposes. Meanwhile, 30% correctly defined biospecimens as being samples and/or biomolecules with annotated clinical, socioeconomic and lifestyle data. A high percentage of students (78%) understood that donating biospecimens to biobanks would require signing a consent form, 57% knew that their data would be kept confidential, and 65% knew that there are SOPs for handling their donated biospecimens.

**Table 2:** Knowledge about biobanking

Knowledge of Biobanking	*N*	%
Aware of the "Human Genome Project"	257	44
Aware of the term "Biobank"	157	27
The purpose of biobank is to collect & store biospecimens for diagnosis, treatment and research purposes	339	59
According to modern biobanking, biospecimens are samples and/or biomolecules with annotated clinical, socioeconomic and lifestyle data	173	30
Donating a biospecimen to a biobank requires signing a consent form	448	78
There is a standard operating procedure (SOP) for biobanks to collect, process, store and release biospecimens	373	65
Biospecimen annotated data will be kept be confidential and anonymous	325	57
Biobanking knowledge score (mean ± SD)	3.2 ± 1.6	

The majority of the students were willing to donate biospecimens to biobanks for biomedical research purposes (89%). The associations between different variables and the willingness to donate biospecimens to biobanks are summarized in Table 3. Factors that were significantly associated with the willingness to donate included marital status (*p*-value = 0.037), faculty (*p*-value < 0.001), general health status (*p*-value = 0.048), past experience with tissue testing and tissue donation (*p*-value = 0.042 and *p*-value < 0.001, respectively), knowledge of biobanking scores (*p*-value < 0.001) and biomedical research attitude scores (*p*-value < 0.001). Factors such as gender, GPA, previous hospitalization, previous blood tests and blood donation were not significantly associated with the students’ willingness to donate biospecimens.

**Table 3:** The associations between socio-demographic, health-related and biobanking-related variables and willingness to donate

	Willingness to donate				*p*-value*
Variables	Yes		No		
	N	%	N	%	
Total	517	89	66	11	---
Socio-demographic & health-related					
Gender					
Male	199	87	30	13	0.281
Female	317	90	36	10	
Marital Status					
Married	57	81	13	19	0.037
Non-married	459	90	52	10	
Faculty					
Medicine	193	85	35	15	**<0.001**
Dentistry	107	92	9	8	
Medical technology	157	96	7	4	
Pharmacology	60	80	15	20	
GPA					
A	167	91	16	9	0.394
B	256	88	34	12	
C/D	67	86	11	14	
General Health					
Excellent	164	91	16	9	**<0.001**
Very good	200	91	20	9	
Good	139	87	21	13	
Fair/Poor	12	60	8	40	
History of Chronic disease					
No	470	89	58	11	0.554
Yes	44	86	7	14	
Family history of inherited disease					
No	330	89	39	11	0.604
Yes					
Previous hospitalization					
No	331	89	40	11	0.651
Yes	183	88	25	12	
Biobanking-related					
Previous genetic testing					
No	493	89	59	11	0.096
Yes	23	79	6	21	
Previous blood testing					
No	45	88	6	12	0.887
Yes	472	89	59	11	
Previous tissue testing					
No	437	90	49	10	**0.042**
Yes	76	83	16	17	
Previous blood donation					
No	370	89	45	11	0.779
Yes	144	88	19	12	
Previous tissue donation					
No	506	90	59	10	**<0.001**
Yes	8	62	5	39	
Involvement in medical research					
No	281	88	38	12	0.495
Yes	231	90	26	10	
Biobanking knowledge score (mean ± SD)	3.17 ± 1.6		2.33 ± 1.6		**<0.001**
Biomedical research attitude score (mean ± SD)	38.1 ± 3.7		35.6 ± 3.8		**<0.001**

*Chi-squared test was used except for biobanking knowledge and biomedical research attitude scores where a *t*-test was used. Correlations with *p*-value < 0.05 are considered statistically significant (﻿Bold font)

Regarding the primary recorded reason for willingness/unwillingness to donate biospecimens to biobanks, participants believed that donations would advance medical research and benefit society (44%) (Table 4). Other reasons included the possibility of notification about abnormal results (25%), benefiting them and their families (15%), biobanks have already been established in developed countries (12%), and that samples will already be collected anyway for diagnostic purposes (10%). The main reasons for unwillingness to donate were concerns about misuse of the biospecimens (15%), followed by fear of needles/injections (13%) and confidentiality concerns (12%). Other reasons included fear of discovering genetic predispositions (10%), concern that genetic information may be used for discrimination (7%), concern that biospecimens may be used for commercial purposes (6%) and religious reasons (3%).

**Table 4:** Reasons for willingness or unwillingness to donate

Reasons for willing to donate	N	%
The biobank will advance medical research, benefit the society and future generation	250	44
My family and I will benefit	84	15
I could be notified about abnormal results	143	25
Samples will already be collected as part of my medical care	55	10
Biobanks are already established as core facility of biomedical research in developed countries	68	12
Reasons for not willing to donate	N	%
Concern about misuse of biospecimen in biomedical research	88	15
Concern about discovering genetic predispositions to some diseases	60	10
Concern about confidentiality	71	12
Concern that genetic information may be used for discriminatory purposes	41	7
Concern that biospecimen may be used for commercial purposes	35	6
Fear of needles/injections	76	13
Religious reasons	16	3

**Fig. 1:**
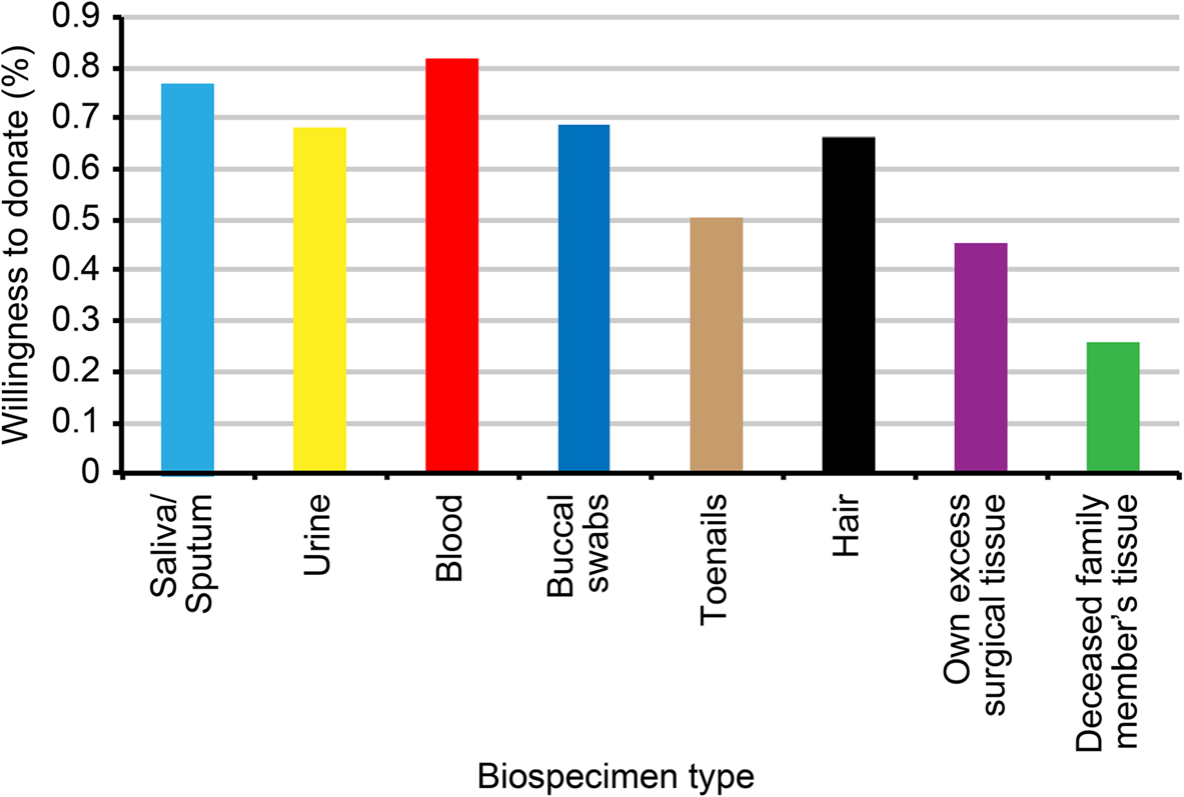
Willingness to donate specific tissue

The tissues that students were willing to donate were mainly blood (82%) and saliva/sputum (77%). A considerable number of students were also willing to donate urine (70%), buccal swabs (66%), hair (67%) and toenails (49%). Despite the fact that the tissue was already designated for removal, only 43% of students were willing to donate their own excess tissue. A deceased family member’s organs or tissues were the least desired for donation (25%) (Fig. 1).

## Discussion

PM primarily pertains to the identification of diagnostic strategies and the tailoring of therapeutic options customized to each individual according to their genetic background. However, the effectiveness of PM depends not only on the genomic signature of the patient but also on our understanding of the different interactions of genomic makeup with biology, lifestyle, wellbeing, and the overall environment. In this context, the emergence of biobanks as networks of biospecimen collections associated with relevant patient information is a cornerstone to the first decoding of human diseases at the molecular level and then refining our understanding of them using individual biodata. Elucidating this complex matching between biospecimens, patient information and OMICs-based data will ultimately help PM improve the current healthcare services offered to patients worldwide. Therefore, the education and awareness of healthcare providers are paramount to ensuring appropriate high-quality biospecimen management and real-time patient information collection according to the latest SOPs. Additionally, broad awareness campaigns among the public regarding the role and functions of biobanks can reinforce these worldwide efforts to deliver more efficient and precise care. An initial assessment of the current knowledge and attitudes about biobanks in a targeted geographical area is a prerequisite to tailoring suitable awareness programmes for the public or general healthcare providers. This type of assessment among senior healthcare students - who are future healthcare providers - is lacking in Saudi Arabia. Therefore, the objective of this questionnaire-based study was to assess the knowledge of senior healthcare students about biobanks, their willingness to donate biospecimens and the predictors of these attitudes to implement appropriate education and training programmes. The questionnaire was customized to collect students’ biodata, health status, knowledge about biobanks and biospecimens, and willingness to donate biospecimens for biobanks and biomedical research.

The analysis indicated that students lacked sufficient knowledge about biobanks, as 73% of these future healthcare providers had never heard of the term “biobank”. Moreover, 56% of the students had never heard of the HGP – the largest megaproject in biology in the last century – which has laid the foundation for several international collaborative and multidisciplinary initiatives to date [27].

Interestingly, and despite the noticeable lack of knowledge about the HGP and biobanks, our findings demonstrated that 89% of students were willing to donate biospecimens to biobanks. This higher enthusiasm of senior healthcare students at KAU to donate biospecimens is in agreement with previous findings from a local study conducted in Riyadh city (Saudi Arabia) and another survey conducted among the general public in Jordan, a neighbouring country with a similar culture [28]. Another study was conducted with patients, who were expected to be less aware of the HGP and biobanking; however, 70% of participants were willing to donate specimens [9]. Similar percentgaes of willingness to donate biospecimens (69%) were reported in two different studies conducted in the USA, one with patients and the other with the Chinese American community [14, 29].

Several studies have investigated factors related to the willingness to donate to biobanks. Our data analysis indicated that such biospecimen donation willingness was significantly associated with several factors, mainly general health, marital status, faculty, past experience with tissue testing and/or tissue donation, biomedical attitude scores and knowledge of biobanking. In fact, we found that better self-reported health status was significantly associated with willingness to donate, and this finding is consistent with similar results reported from potential sample donors amongst the Swedish general population [7]. In addition, previous tissue tests and tissue donations were positive significant predictors in our study, with more than 62% of respondents being willing to donate their own future “extra” surgical tissue following the completion of all required medical diagnostics and therapeutics. However, in another local study targeting Saudi outpatients with a previous history of tissue tests and/or donation, the percentage willing to donate biospecimens was only approximately 9% [9]. Compared with our results, this conspicuous difference might be because our cohort of interviewees consisted of senior healthcare students, who were therefore aware of the importance of voluntary tissue donations for the enhancing both biomedical research and clinical practice.

To ensure suitable decision-making and customized awareness in healthcare students, an assessment of their knowledge and attitudes about biobanks and biomedical research was necessary [30]. Therefore, our questionnaire was designed to facilitate a better understanding of the factors influencing their attitudes and to transform their knowledge and willingness into measurable scores. In fact, the findings of the current study revealed that student knowledge of biobanking and biospecimens was insufficient, identifying a mean knowledge score of 3.6 out of 7. Approximately 59% correctly defined the purpose of biobanks, and 30% were able to guess the correct definition of biospecimens. This knowledge score was expected, as less than half of students had heard about the HGP, and approximately one third had heard of the term “biobank”. In contrast, a study conducted among members of Kaiser Permanente in Colorado members who were approached in clinical waiting rooms found that the majority (85%) of these patients – in contrast to our cohort composed of senior medical students – answered correctly when they were asked about general information regarding biospecimen collection for research purposes. This high percentage was attributed to the brochures and a draft consent form that were distributed to the respondents prior to answering the survey [14].

Measuring general attitudes towards biomedical research has also been found to significantly predict willingness to participate in biomedical research and donate biospecimens for research [26]. A Swedish study demonstrated that a positive public attitude towards genetic research was significantly associated with willingness to donate for research purposes [31]. An Italian survey reported that people who were willing to donate samples had a more positive attitude towards biomedical research than those who were not willing to donate [8]. Kobayashi et al. found that a positive attitude towards pharmacogenomics research was significantly associated with willingness to donate samples for biobanking [32]. Interestingly, the HGP outcomes and biobanking initiatives were shown to reinforce positive attitudes towards genetics and a greater willingness to donate biospecimens, as reflected in the Dutch public opinion in 2010 compared with that in 2002. The respondents exhibited higher expectations of biomedical research and thought that “genetic testing should be promoted more intensively” [33].

Regarding the factors related to willingness to donate biospecimens, our study demonstrated that participants’ belief that “biobanks will advance medical research and benefit society and future generations” was the major factor influencing this willingness. The high biomedical research attitude score (38 out of 40) identified in this study was consistent with several local [34] and worldwide [7, 8, 14, 33, 35] studies. A study conducted with Michigan college students found that many of them were very supportive of donating, hoping that it would benefit future patients [36]. The main reasons participants in this study were not willing to donate were concerns about misuse of biospecimens, worries about confidentiality and fear of needles/injections. Concerns about confidentiality have also been stated to be a main reason for refusing to donate in other studies elsewhere [14, 29] and were highlighted by other scientists and medical healthcare providers as evidence of the importance of building “trust” with the public [30, 37–41]. In a conservative society, it was worth investigating the impact of religious beliefs, which were surprisingly reported to be the main reason for not willing to donate biospecimens by only 3% of the respondents. This contribution of religious beliefs in the decision to donate was lower than the percentage identified in another local study in 2009 targeting University students. This former study showed that approximately two-thirds of students linked their decisions to donate deceased relatives’ tissues and organs with a jurisprudential opinion issued by religious experts/scholars [22]. This difference might be explained by the fact that the current study targeted senior students in the biomedical field. Additionally, the previous study was performed 7 years ago, and we think that since then, an important focus on genetics through social media has increased awareness of the importance of genetics and biomedical research among students attending KAU in 2016. However, this awareness remains too general and lacks the guidance and depth to grasp the importance of biobanking in the post-genomic era. A slight regional effect between Saudi Arabian regions might be involved, as suggested elsewhere [9]. Other studies in neighbouring countries have reported that 16% of medical students in Turkey were not willing to donate biospecimens for religious reasons, whereas 61% of the Jordanian public correlated their biospecimen donations with religious permission [23, 42].

In concordance with our findings, several studies have reported that scores for both biobanking knowledge and biomedical research attitudes were significantly higher in people willing to donate for research purposes than in those who were not. Goddard and colleagues in 2009 reported that participants who were willing to donate to biobanks were more likely to correctly answer knowledge questions [29], which supports our data from senior medical students. Level of education has also been significantly positively correlated with willingness to participate in biobanking specimen collection in Jordan [28].

This assessment of the current status of knowledge about biobanking and biomedical research sheds light on the necessity and opportunity to establish more personalized education and awareness strategies about PM and biobanking in the post-genomic era. An urgent revision of academic programmes delivered to medical students is needed to adopt the Double Helix Curriculum (DHC) by integrating of basic sciences and clinical biomedicine. Medical schools should also consider including teaching of advanced research methodology and OMICs-based technologies in their curricula [43]. More active involvement of students in biomedical research activities should be highly recommended and credited [30]. In parallel to this effort, additional awareness campaigns designed to raise public awareness about PM and biobanking and to highlight the importance of involvement are essential to building trustworthy partnerships [37, 44] for more effective biobank establishment and governance. This type of awareness programme is essential among healthcare providers and all biobanking stakeholders to bridge the gap between clinicians and scientists [45]. Therefore, deliberate policies and guidelines that will foster state-of-the art research in biomedical and clinical environments could be implemented to expedite the transition towards PM [46].

Several studies have reported significant effects of some of the predictors of willingness to donate biospecimens that were not significant in our survey. These factors include previous participation in medical research [9, 18], gender [29], age [7, 14, 16–19] and a history of previous hospitalization [9]. In addition to possible regional and cultural effects, these discrepancies between our results and the aforementioned previous reports targeting the general public (heterogeneous population) could be attributed to our population of interviewees, whio were exclusively senior health care students. In fact, the selection criteria stringency adopted in our questionnaire generated a sample of relatively homogenous individuals receiving similar curricula with comparable previous participation in medical research.

Regarding students’ willingness to donate specific tissues, relatively high willingness rates were recorded for blood (82%), saliva/sputum (77%), urine (70%), buccal swabs (66%), hair (67%) and toenails (49%) (Figure 1). Willingness to donate was highest for blood donations, which was comparable to findings of other local studies [9]. This high percentage of blood donation willingness may be due to this population’s familiarity with the concept of blood donation. In contrast, only about half the students in this study were willing to donate their own excess surgical tissue (43%), which was lower than percentages in previous reports in which approximately 70% of both Swedish and Saudi respondents from the general public agreed to donate excess surgical tissue [9, 47]. This finding was unexpected, especially from senior healthcare students who knew that these collected tissues would serve diagnostic and therapeutic purposes; only leftover tissue may be used for biomedical research following informed consent. This result could be due to the students’ education level, which is associated with higher awareness of bioethics, confidentiality and possible misuse, as approximately 27% justified their attitude with concerns about confidentiality (12%) or biospecimen misuse (15%). These results highlighted an important issue related to the lack of biobanking knowledge and trust between stakeholders, including clinicians [37, 48]. Therefore, creating future biobanking structures requires a comprehensive strategy that fosters trust between the public, healthcare providers, and policy makers to bridge the gap between scientists and clinicians and improve the welfare of the Saudi population. Other respondents reported concerns about discovering genetic predispositions to some diseases (10%), and these concerns could be influenced by the relatively high rate of consanguinity in Saudi Arabia [49, 50].

Only a quarter of students were willing to donate a deceased family member’s organs, and this percentage was comparable to that of other local studies targeting patients [9, 34]. These local willingness percentages were lower than those reported worldwide. For example, a study on medical students in Pakistan reported that 45% were willing to donate their organs for transplantation [51]. Similar rates (48%) were reported in the Swedish public, who stated that they would be prepared to allow a member of a research ethics committee to make decisions about the use of their own tissue when they weredeceased [47].

## Conclusion

Since the publication of the first version of the human genome sequence, tremendous efforts have been made by the scientific community worldwide to uncover the genomic aetiology of diseases and to identify robust actionable and druggable targets for individualized therapeutics. These achievements have been attained thanks to the billions of biospecimens managed by biobanks in addition to the unprecedented outreach, education and networking amongst all stakeholders. The establishment of such an expanding international network was preceded by several comprehensive awareness programmes and workshops to foster the transition towards personalized healthcare [15]. Nevertheless, this transition revealed unexpected levels of genomic complexity that required additional international collaborative efforts along with broader awareness campaigns. The current study was designed to address this awareness gap in the MENA (Middle-East and North-Africa) region, specifically in Saudi Arabia, through a customized questionnaire targeting senior healthcare students. The results indicated a noticeable lack of knowledge about the HGP and biobanking amongst the respondents. However, the students expressed a high willingness to donate a wide range of biospecimens for biomedical research, and this willingness was significantly influenced by their knowledge of the HGP and biobanking scores, past experience with tissue test and/or tissue donation, biomedical attitude scores, and health status. Additional awareness and educational programmes tailored to these future healthcare providers to familiarize them with biobanking, biospecimen donation, genomics technologies and their scope in clinical applications are a paramount to helping students adhere to the PM international movement. Such awareness about the importance of informative involvement in biobanking, biospecimen donation and OMICs-driven medicine should also be expanded to all biobanking staff members, healthcare providers and the general public to foster state-of-the art biomedical research and to deliver clinically relevant applications that enhance the healthcare system.

## Abbreviations

GPA: Grade-point average
HGP: Human genome project
KAU: King Abdulaziz University
PM: Precision medicine
RAQ: Research attitudes questionnaire
SOPs: Standard operating procedures
